# Exosome-like nanovesicles derived from Phellinus linteus inhibit Mical2 expression through cross-kingdom regulation and inhibit ultraviolet-induced skin aging

**DOI:** 10.1186/s12951-022-01657-6

**Published:** 2022-10-21

**Authors:** Jingxia Han, Ting Wu, Jing Jin, Zhiyang Li, Wenjun Cheng, Xintong Dai, Kai Yang, Heng Zhang, Zhiyuan Zhang, Haohao Zhang, Rong Fan, Shaoting Zheng, Haoyang Liu, Yinan Li, Huan Zhao, Cheng Yao, Tingting Lin, Caibin Zhu, Huijuan Liu

**Affiliations:** 1 Cheermore Cosmetic Dermatology Laboratory, Shanghai, China; 2 State Key Laboratory of Medicinal Chemical Biology and College of Pharmacy, Nankai University, Tianjin, China; 3 Medical Plastic and Cosmetic Center, Tianjin Branch of National Clinical Research Center for Ocular Disease, Tianjin Medical University Eye Hospital , Tianjin, China; 4 CanSino Biologics Inc, Tianjin, China

**Keywords:** Skin aging, Fungi exosome-like nanovesicles, miRNAs, Anti-aging effects, Cross-kingdom regulations

## Abstract

**Background:**

*Phellinus linteus* (PL), which is a typical medicinal fungus, has been shown to have antitumor and anti-inflammatory activities. However, studies on the effect of anti-photoaging are limited. Studies have shown that exosome-like nanovesicles are functional components of many medicinal plants, and miRNAs in exosome-like nanovesicles play a cross-kingdom regulatory role. At present, research on fungi exosome-like nanovesicles (FELNVs) is few.

**Results:**

We systematically evaluated the anti-aging effects of PL. FELNVs of PL were isolated, and the functional molecular mechanisms were evaluated. The results of volunteer testing showed that PL had anti-aging activity. The results of component analysis showed that FELNVs were the important components of PL function. FELNVs are nanoparticles (100–260 nm) with a double shell structure. Molecular mechanism research results showed that miR-CM1 in FELNVs could inhibit Mical2 expression in HaCaT cells through cross-kingdom regulation, thereby promoting COL1A2 expression; inhibiting MMP1 expression in skin cells; decreasing the levels of ROS, MDA, and SA-β-Gal; and increasing SOD activity induced by ultraviolet (UV) rays. The above results indicated that miR-CM1 derived from PL inhibited the expression of Mical2 through cross-kingdom regulation and inhibited UV-induced skin aging.

**Conclusion:**

miR-CM1 plays an anti-aging role by inhibiting the expression of Mical2 in human skin cells through cross-species regulation.

**Graphical abstract:**

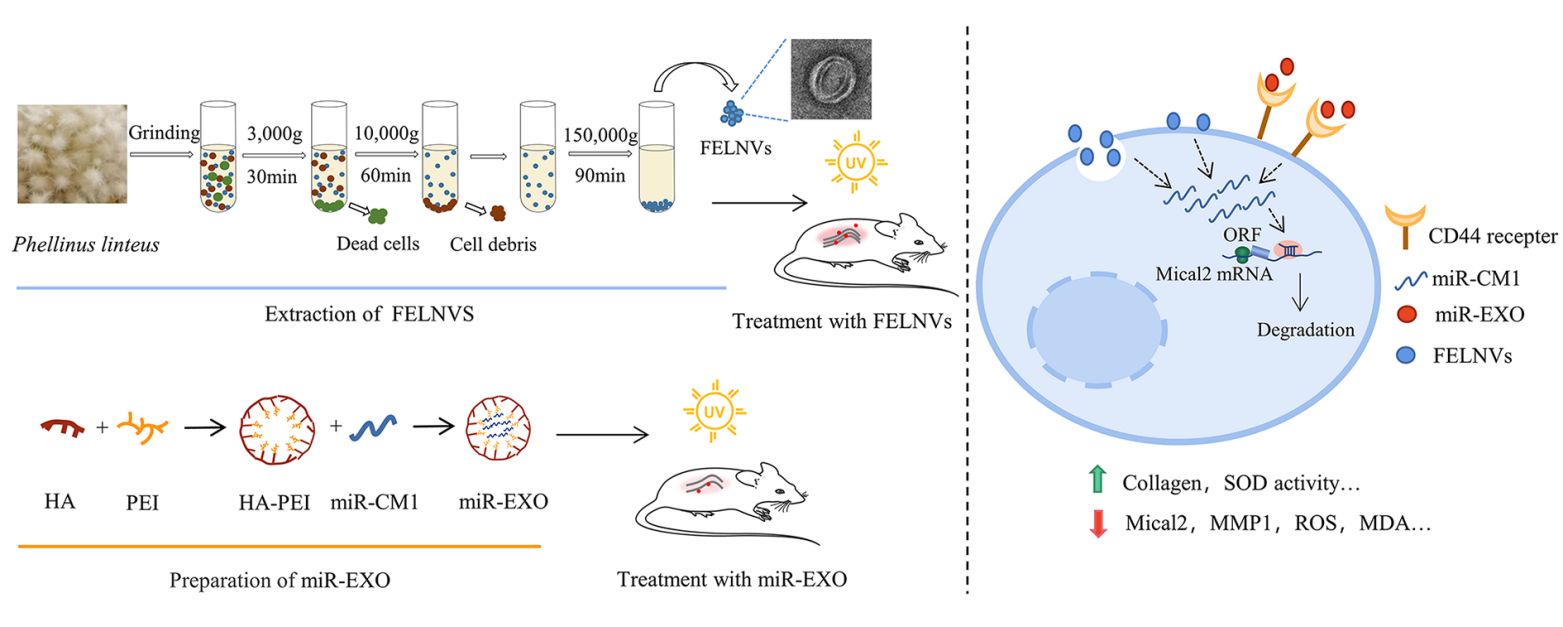

**Supplementary Information:**

The online version contains supplementary material available at 10.1186/s12951-022-01657-6.

## Background

The skin is the body’s first line of defense and is most susceptible to damage and aging caused by the influence of the external environment, such as ultraviolet (UV) radiation, air pollutants, cold, and wind. Among them, the most important factor inducing skin aging is UV irradiation [1]. It is also called photoaging due to UV radiation from the sun and is characterized by poor epidermal keratosis with abnormal hyperplasia, reduced collagen, loss of elasticity, wrinkles, and so on [2]. UV exposure can cause the expression of matrix metalloproteinases (MMPs) in the dermis (the site of photodamage), which in turn degrade dermal collagen [3]. In addition, UV can mediate inflammation through reactive oxygen species (ROS) and lead to aging [4]. Therefore, the search for active molecules that can alleviate photoaging is of great significance for the development of drugs and skin care products.

Phellinus linteus (PL) is a typical medicinal fungus containing polysaccharides, polyphenols, flavone, and other compounds, which have been proved to play a significant regulatory role in anti-tumor function, hepatoprotection, reduction in inflammation, and blood glucose control [5–8]. For example, polysaccharides isolated from PL can inhibit the MAPK and PPAR pathways to inhibit the expression of various inflammatory factors in cells [9]. In addition, the water-soluble extract from PL exerts immunomodulatory effects in atopic dermatitis [10]. Its extract has now been used in the formulation of various skincare products and cosmetics, but the molecular mechanisms at the skin level are not well understood. Elucidation of the molecular mechanism by which PL functions will further provide data support for its application.

Exosomes are one of the extracellular vesicles secreted by eukaryotic cells, ranging from 40 mm to 160 nm in size with an endosomal origin [11, 12]. Following the discovery of exosomes in mammalian cells, accumulating evidence indicates that exosome-like vesicles occur in plants, which are termed plant exosome-like nanovesicles (PELNVs). PELNVs are 50–500 nm in size and similarly contain lipids, proteins, and nucleic acids, which have antitumor function and regenerative effects; regulate intestinal diseases; and provide hepatoprotection [13–15]. Ginger-derived nanoparticles can induce liver detoxification and antioxidation and protect mice from alcohol-induced liver injury [16]. Grapefruit-derived nanoparticles can act as an immune modulator in the intestine and attenuate inflammatory responses in colitis [17]. For fungi exosome-like nanovesicles, studies have focused on extracellular vesicles (EVs) of pathogenic fungi and yeast, and fungal EVs play a role in cellular metabolism, signal transduction and virulence [18, 19]. Several pathogenic fungi have been reported to be shown to produce EVs containing various components associated with virulence, such as Cryptococcus neoformans EVs containing enzymes that catalyze melanin synthesis, which guarantees their virulence and survival [20]. However, the exosome-like nanovesicles in PL have not been studied.

Exosome-like nanovesicles of PL have not been characterized and studied so far. Whether PL can exert cross-kingdom regulatory functions through miRNAs also requires further exploration. Therefore, in this work, we explored the efficacy and molecular mechanisms of PL in resisting skin photoaging. We discovered and characterized nanoparticles secreted by PL, which we termed fungi exosome-like nanovesicles (FELNVs), and continued to explore their anti-aging molecular mechanism.

## Results

### PL extract can effectively improve the skin of volunteers

**Fig. 1:**
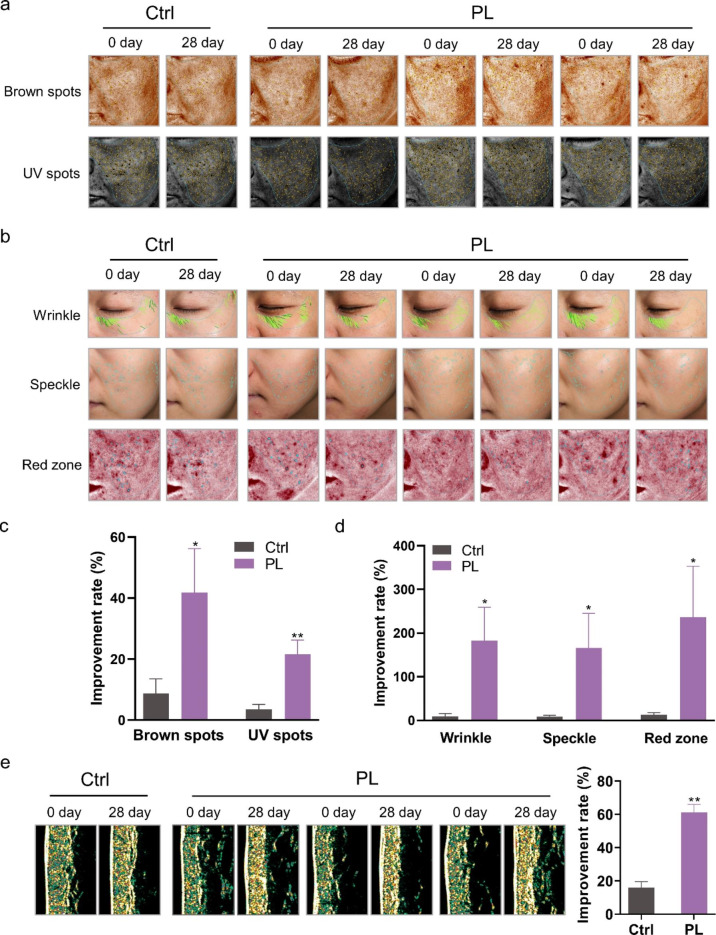
**Effect of PL extract on the skin of volunteers. a, b** Skin image detected by VISIA imaging system showing the brown spots, UV spots, wrinkles, speckles, and red zone of volunteers. **c, d** Statistical analyses of the improvement rate of brown spots, UV spots, wrinkles, speckles, and red zone. **e** Collagen density in the arm of volunteers detected by the Dermalab instrument. Data are expressed as mean ± SD (*P < 0.05, **P < 0.01)

PL extract is listed in the “catalogue of used cosmetic raw materials” (version 2021, China). We used PL on volunteers’ facial skin and arm to test its efficacy. The indexes of facial skin were detected and recorded over a period of 28 days by the VISIA imaging system (Fig. 1a and b). Results showed that the brown spots, UV spots, wrinkles, speckles, and red zone improved remarkably in the volunteers treated with PL. Compared with the control group, the brown spots and UV spots of the PL-treated group were reduced, indicating that PL displayed a significant improvement in the pigmentation caused by UV irradiation (Fig. 1a and c). The percentage of wrinkles, speckles, and red zone in the PL-treated group significantly improved, indicating that PL may be beneficial in delaying aging and reducing the potential inflammatory reaction of volunteers’ skin (Fig. 1b and d).

Dermalab combo is used to detect skin physiological indexes through the connecting probe, in which a high-frequency skin ultrasound probe can quickly measure skin structure, such as skin thickness and collagen density. We examined the collagen density of the volunteers’ arms before and after the use of PL to evaluate the anti-aging effect of PL. The results showed that the increase in collagen density after PL treatment was higher than that in the control group (Fig. 1e). Thus, PL demonstrated good anti-aging and anti-inflammatory effects on the volunteers.

### Extraction and identification of FELNVs from PL

**Fig. 2:**
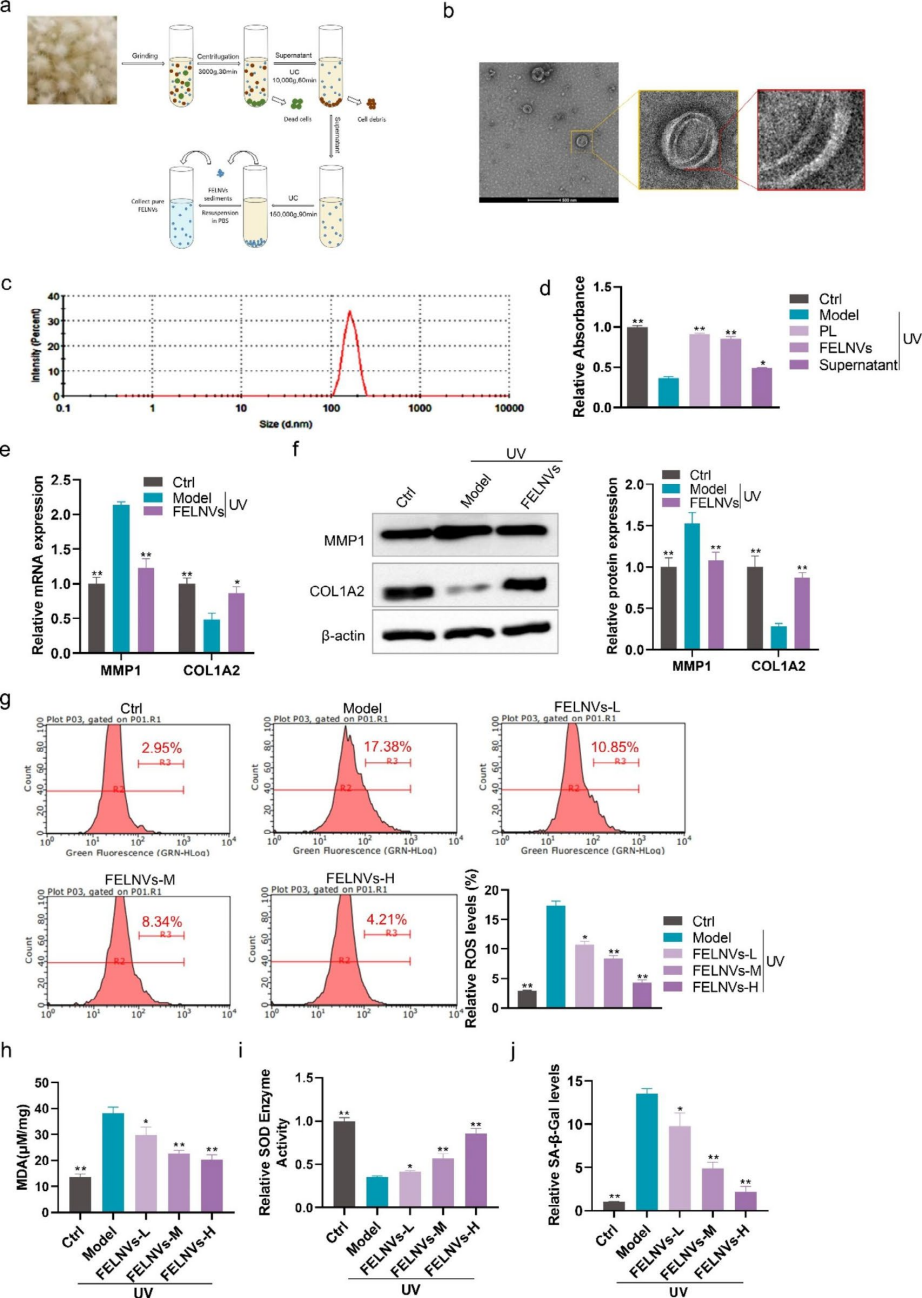
**FELNVs can resist UV-induced aging in HaCaT cells. a** Flow diagram to show FELNV extraction using differential ultracentrifugation. **b** Representative TEM image of purified FELNVs from PL. Scale bar, 500 nm. **c** Average size of FELNVs was characterized by NanoSight analysis. **d** Effect of PL extract (PL), FELNVs, and supernatant on proliferation of UV-treated HaCaT cells by CCK8 assay. Model: UV-treated. **e** Effect of FELNVs on mRNA expression levels of MMP1 and COL1A2 in UV-treated HaCaT cells tested by qRT-PCR. **f** Effect of FELNVs on the expression of MMP1 and COL1A2 in UV-treated HaCaT cells tested by Western blot. **g–j** Relative ROS levels (g), MDA content (h), relative SOD enzyme activity (i), and relative SA-β-Gal levels (j) in UV-treated HaCaT cells with FELNVs treatment at different concentrations. Data are expressed as mean ± SD (*P < 0.05, **P < 0.01)

To explore which component of PL works, we used differential ultracentrifuge (UC) techniques to obtain the whole PL extract, FELNVs, and supernatant (Fig. 2a). The results of NanoSight analysis showed that the diameters of FELNVs ranged from 100 to 260 nm (Fig. 2c), which was different from the reported mammalian exosome size of 40–160 nm but consistent with the size of PELNVs from 50 to 500 nm. We also excluded the interference of spores because overall mushroom spore dimension is at the micron level [21, 22]. FELNVs have a “double-layer shell” structure detected by transmission electron microscopy (TEM; Fig. 2b), and this unique structure may provide them with improved stability and load-bearing capacity.

### FELNVs can inhibit UV-induced aging in HaCaT cells

We further explored which component of PL plays a major role. CCK8 assay was used to test the effects of different components of PL on the proliferation of HaCaT cells. After 24 h of pre-protection by PL, FELNVs or supernatant, UV-induced cell viability damage can be relieved in different degrees, and the effect of FELNVs was better than that of water-soluble small molecule active substance in supernatant (Fig. 2d). To investigate whether there is a change in senescent markers with FELNV pre-protection, we examined the expression of MMP1 and COL1A2, which are markers of senescence, in the UV-induced photoaging model. MMP-1 can degrade type I and type III collagen and destroy the normal structure of collagen fibers and elastic fibers when overexpressed [3]. Compared with the model group, the mRNA and protein expression levels of MMP1 in the FELNVs-treated group were reduced, while the expression of COL1A2 increased (Fig. 2e and f).

The generation of large amounts of ROS by UV irradiation can exacerbate oxidative stress damage including lipid oxidation, thereby leading to human skin cell senescence; one of the products of lipid oxidation is malondialdehyde (MDA), which can indirectly assess oxidative stress [23, 24]. Superoxide dismutase (SOD) is an antioxidant metalloenzyme that scavenges oxygen radicals [25]. In addition, senescent cells often become larger in size and express senescence-associated β-galactosidase (SA-β-Gal), which is a biomarker of skin aging [26]. Thus, we further detected the effects of FELNVs on the ROS level, MDA content, SOD enzyme activity, and SA-β-Gal level (Fig. 2 g-j). FELNV pre-protection could reduce levels of ROS, MDA, and SA-β-Gal and increase SOD enzyme activity in HaCaT cells in a dose-dependent manner (Fig. 2 g-j). These results showed that FELNVs of PL exerted anti-aging effects.

### RNAs in FELNVs play a major anti-aging role

**Fig. 3:**
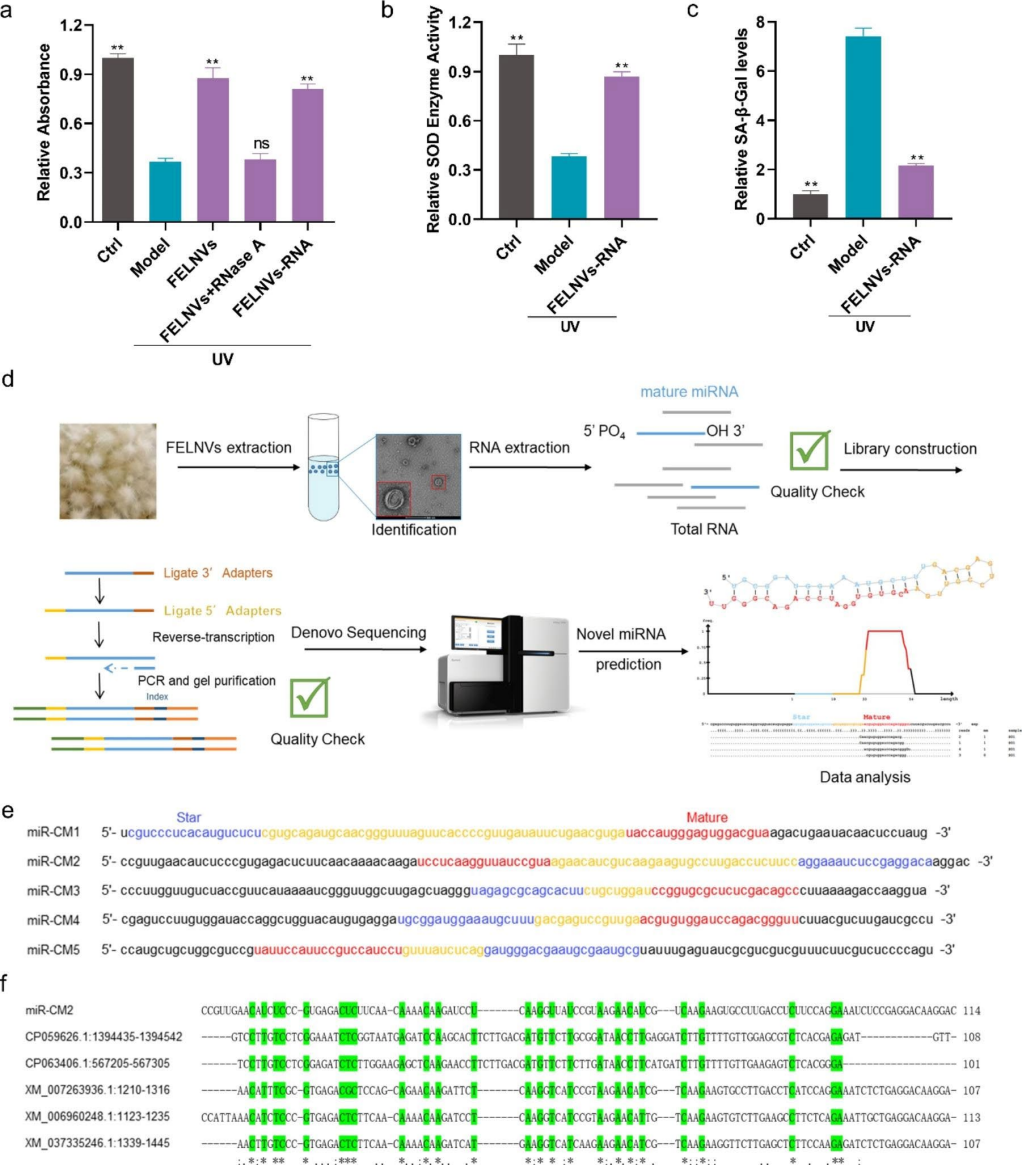
**RNAs in FELNVs exert anti-aging effects. a** Effect of FELNVs, RNA free components (FELNVs + RNase A), and RNA components (FELNVs-RNA) on proliferation in UV-treated HaCaT cells by CCK8 assay. **b, c** Relative SOD enzyme activity (b) and relative SA-β-Gal levels (c) in UV-treated HaCaT cells with RNA treatment of FELNVs. **d** Flow diagram to show de novo miRNA sequencing and prediction of novel miRNAs. **e** Sequences of five novel miRNAs (miR-CM1 to CM5) of FELNVs. **f** Sequence alignment of miR-CM2 with nucleotide collection (nr/nt) of NCBI. Data are expressed as mean ± SD (*P < 0.05, **P < 0.01)

We also explored the effects of components in the FELNVs of PL, which play a major role in anti-aging. The effect of FELNVs, RNA-free components (FELNVs + RNase A), and RNA components (FELNV-RNA) on proliferation in UV-treated HaCaT cells was determined by CCK8 assay. HaCaT cells were pre-protected with the above three components. The results showed that both FELNVs and FELNVs-RNA groups could protect HaCaT cells from UV damage, and the cell viability was higher than that of the model group. There was no significant difference in cell viability between the FELNVs + RNase A group and model group (Fig. 3a). Further detection revealed that the FELNVs-RNA could significantly increase SOD enzyme activity and reduce SA-β-Gal levels compared with the model group (Fig. 3b and c). These results showed that RNA in FELNVs played a major anti-aging role.

### miRNA sequencing and prediction of novel miRNAs

**Fig. 4:**
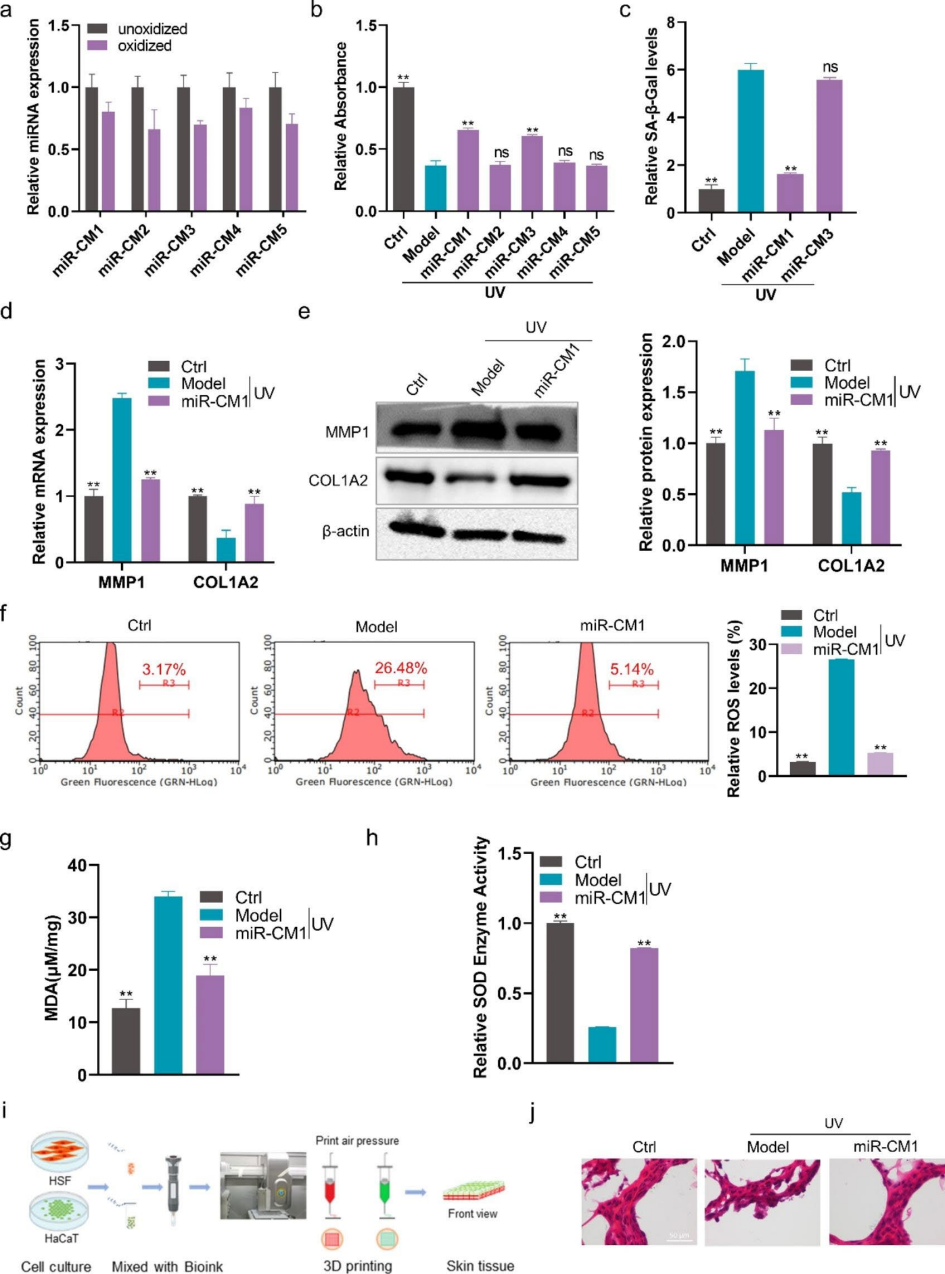
**miR-CM1 in FELNVs exert anti-aging effects. a** Comparison of expression levels of the five novel miRNAs of FELNVs with/without oxidation treatment. **b** Effect of five novel miRNAs on proliferation in UV-treated HaCaT cells by CCK8 assay. **c** Relative SA-β-Gal levels in UV-treated HaCaT cells with miR-CM1 or miR-CM3. **d** Effect of miR-CM1 on mRNA expression levels of MMP1 and COL1A2 in UV-treated HaCaT cells was tested by qRT-PCR. **e** Effect of miR-CM1 on the expression of MMP1 and COL1A2 proteins in UV-treated HaCaT cells was tested by Western blot. **f–h** Relative ROS levels (f), MDA content (g) and relative SOD enzyme activity (h) in UV-treated HaCaT cells with miR-CM1 treatment. **i** Flow diagram to show 3D cell printing. **j** Representative imaging of H&E stain of skin tissue from 3D printing and cultures. Data are expressed as mean ± SD (*P < 0.05, **P < 0.01)

Studies have shown that exosomes have a high level of miRNAs, which can participate in post-transcriptional regulation of gene expression and participate in various pathophysiological processes [27–29]. After extracting the RNA from FELNVs of PL, de novo miRNA sequencing was carried out, and the data were analyzed to predict the novel miRNAs (Fig. 3d). Five novel miRNAs were identified, namely, miR-CM1 to miR-CM5 (Fig. 3e). Compared with the nucleotide collection (nr/nt) by Standard Nucleotide BLAST, the sequence of miR-CM2 partially matched with the existing known genome sequence, while miR-CM1/CM3/CM4/CM5 did not overlap with the existing known sequence (Fig. 3f). 2’-O-Methyl modification on plant miRNAs renders them resistant to periodate. These five miRNAs (miR-CM1, miR-CM2, miR-CM3, miR-CM4, and miR-CM5) showed obvious resistance to periodate (Fig. 4a). In summary, the five novel miRNAs were more stable than mammalian-derived miRNAs.

### miR-CM1 pre-protection can resist UV-induced cell senescence through cross-species regulation

To investigate the effects of miR-CM1 to miR-CM5 in the UV-induced photoaging model of HaCaT cells, the five miRNAs were transfected to cells. CCK8 assay showed that miR-CM1 and miR-CM3 pre-protection could inhibit the decrease in cell viability of HaCaT cells caused by UV rays (Fig. 4b). Furthermore, miR-CM1 pre-protection could reduce the level of SA-β-Gal, which had better anti-aging effect than miR-CM3 (Fig. 4c). Therefore, in the following study, we focused on exploring the function and mechanism of miR-CM1 in skin protection. Compared with the model group, miR-CM1 pre-protection could reduce MMP1 expression and increase COL1A2 expression in the UV-induced photoaging model of HaCaT cells (Fig. 4d and e). In addition, miR-CM1 pre-protection could reduce ROS levels and MDA content and improve SOD enzyme activity (Fig. 4f-h). We obtained skin tissues by 3D printing and air–liquid interface (ALI) culture (Fig. 4i). Images of HE staining indicated that the skin structure of the model group was disordered, while the skin structure of the miR-CM1 pre-protection group was normal and close to the skin morphology of the control group (Fig. 4j). In conclusion, miR-CM1 pre-protection could alleviate UV-induced cell senescence.

### Cross-kingdom regulatory mechanisms of miR-CM1

**Fig. 5:**
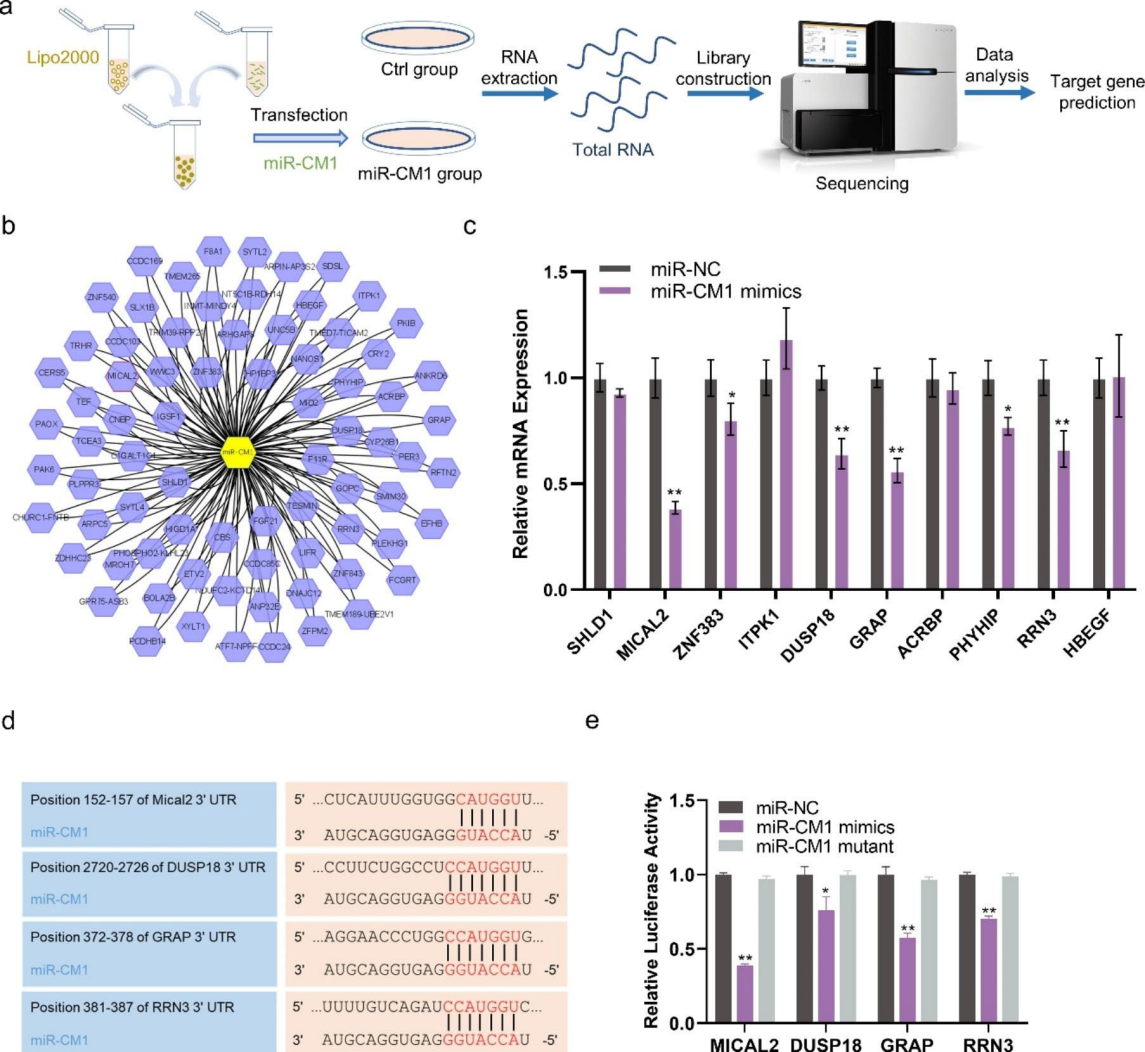
**miR-CM1 has cross-kingdom regulatory activity. a** Flow diagram to show transcriptome Illumina sequencing in HaCaT cells with miR-CM1 transfection. **b** The predicted results of miR-CM1 regulating mRNA. **c** mRNA expression levels of *SHLD1*, *MICAL2*, *ZNF383*, *ITPK1*, *DUSP18*, *GRAP*, *ACRBP*, *PHYHIP*, *RRN3* and *HBEGF* in HaCaT cells with miR-CM1 transfection were tested by qRT-PCR. **d** The matching results between the seed region of miR-CM1 and the 3’UTR region of MICAL2, DUSP18, GRAP and RRN3. **e** Relative luciferase activity of MICAL2, DUSP18, GRAP and RRN3 with miR-CM1 transfection by dual luciferase reporter gene assay. Data are expressed as mean ± SD (*P < 0.05, **P < 0.01)

Chenyu Zhang’s team demonstrated that native plant miRNAs in food can regulate the expression of mammalian target genes [30]. We examined whether miR-CM1 derived from PL can achieve cross-species regulation and play an anti-aging role in a similar way. Illumina sequencing technology was performed on the control group and miR-CM1 transfection group (Fig. 5a). Figure 5b shows the predicted target genes of miR-CM1 (Fig. 5b). Ten genes with high scores were selected from the prediction results to verify the regulatory effect of miR-CM1. qPCR results showed that miR-CM1 mimics could significantly reduce the expression levels of *SHLD1*, *MICAL2*, *ZNF383*, *ITPK1*, *DUSP18*, *GRAP*, *ACRBP*, *PHYHIP*, *RRN3* and *HBEGF* mRNA (Fig. 5c). Among them, *MICAL2*, *DUSP18*, *GRAP* and *RRN3* were significantly down-regulated, so these four were selected for subsequent experiments. Figure 5d shows the results of matching the seed sequence of miR-CM1 with the 3’UTR of MICAL2, DUSP18, GRAP and RRN3 (Fig. 5d). Luciferase report experiments further confirmed that miR-CM1 mimics had obvious regulatory effects on the 3’UTR of MICAL2, DUSP18, GRAP and RRN3 (Fig. 5e). Among them, the regulatory effect on *MICAL2* was the strongest (Fig. 5e). Mical2 is a monooxygenase that directly binds and depolymerizes F-actin, generating ROS in actin microfilament regulation [31–33].

### miR-CM1 inhibits UV-induced aging by inhibiting Mical2 expression

**Fig. 6:**
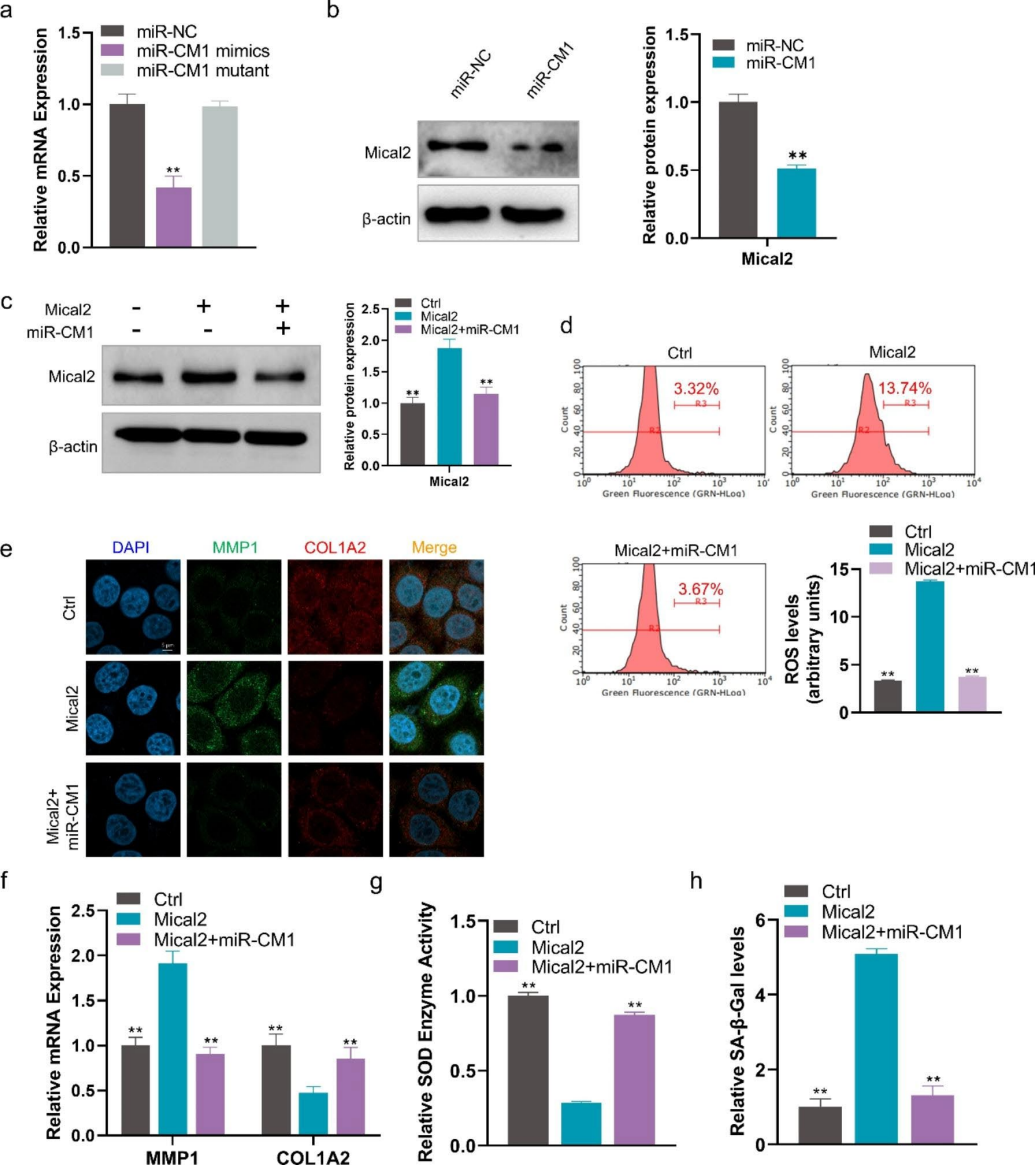
**miR-CM1 resists UV-induced aging by inhibiting Mical2 expression. a** mRNA expression levels of Mical2 in HaCaT cells with miR-CM1 transfection tested by qRT-PCR. **b** Protein expression of Mical2 in HaCaT cells with miR-NC or miR-CM1 transfection were tested by Western blot. **c** Protein expression of Mical2 in HaCaT cells with Mical2 transfection or co-transfection of Mical2 and miR-CM1 were tested by Western blot. **d** ROS levels in HaCaT cells with Mical2 transfection and co-transfection of Mical2 and miR-CM1. **e** Representative imaging of IF in HaCaT cells labeled for MMP1 (green), COL1A2 (red), and nuclear (DAPI: blue) in different treatment groups. **f** mRNA expression levels of MMP1 and COL1A2 in HaCaT cells with miR-CM1 transfection and co-transfection of Mical2 and miR-CM1 tested by qRT-PCR. **g, h** SOD enzyme activity (g) and relative SA-β-Gal levels (h) in HaCaT cells with miR-CM1 transfection and co-transfection of Mical2 and miR-CM1. Data are expressed as mean ± SD (*P < 0.05, **P < 0.01)

To further demonstrate the roles of miR-CM1 in Mical2 regulation, qPCR and Western blot experiments were carried out. The results showed that the mRNA and protein expression levels of Mical2 were significantly downregulated with the transfection of miR-CM1, but no significant difference between the miR-NC group and mutant group was found (Fig. 6a and b). Furthermore, overexpression of Mical2 could increase the protein level of Mical2, while co-transformation of Mical2 and miR-CM1 restored Mical2 expression (Fig. 6c). Subsequent functional experiments showed that overexpression of Mical2 could increase ROS level, MMP1 expression and SA-β-Gal level but reduce the expression of COL1A2 and SOD enzyme activity (Fig. 6d-h). Co-transfection with miR-CM1 could restore these changes in aging-related markers (ROS, SOD, SA-β-Gal, MMP1, and COL1A2) caused by Mical2 overexpression (Fig. 6d-h). These results suggested that overexpression of Mical2 could lead to the emergence of the cell aging phenotype, and miR-CM1 could play an anti-aging role by inhibiting Mical2 expression.

### miR-CM1 encapsulated in artificial exosomes can reduce UV-induced aging in mice

**Fig. 7:**
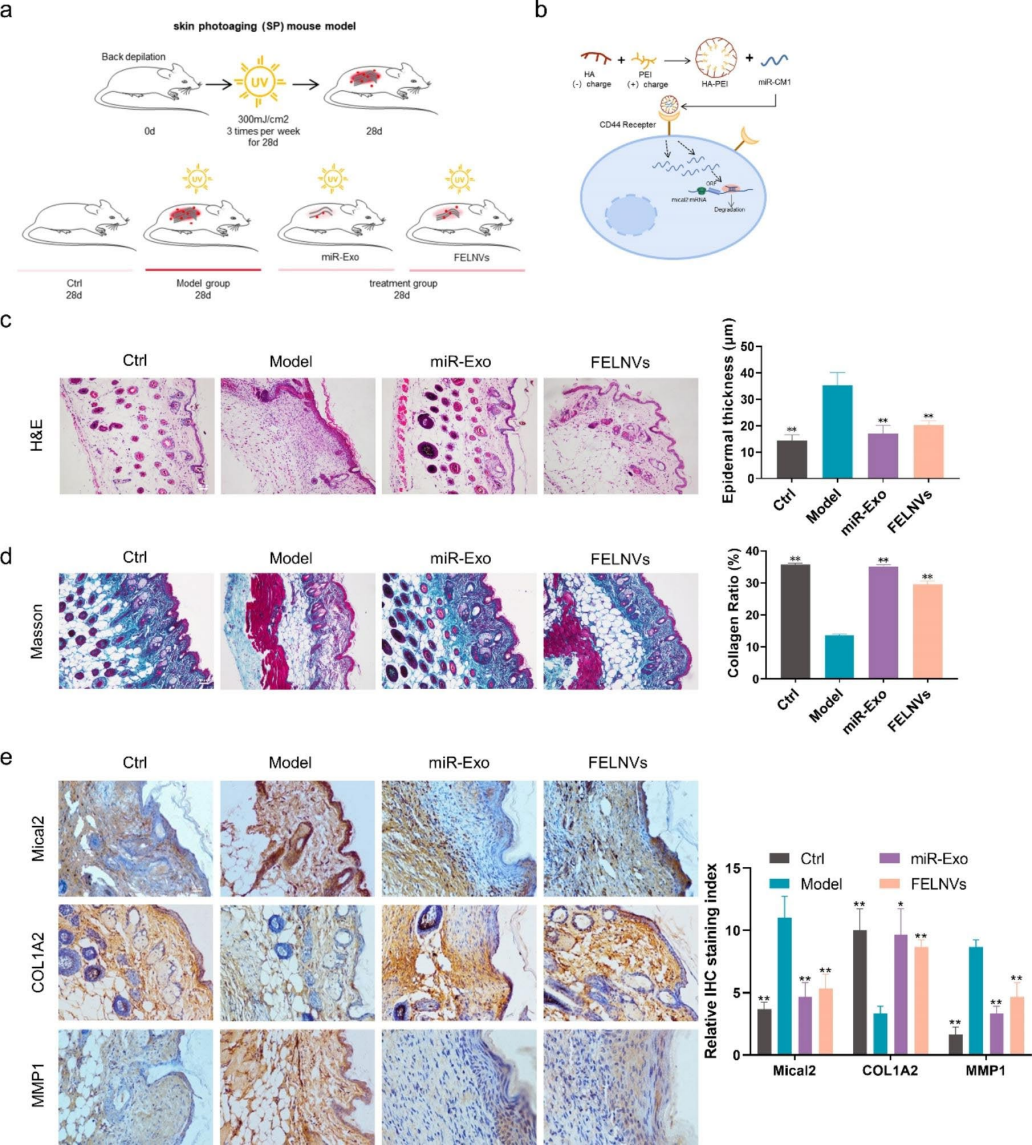
**miR-CM1 encapsulated in artificial exosomes can reduce UV-induced aging in mice. a** Schematic of the mouse photoaging model. Treatment groups were separately given drug treatment of miR-Exo and FELNVs to the dorsal skin of mice before UVA irradiation. **b** Schematic demonstrating miR-CM1 encapsulated in artificial exosomes (EXO mimics) into cells to function. **c-e** Representative images of H&E staining (c), Masson staining (d), and immunohistochemistry (IHC) (e) of skin tissue from mice. Data statistics for epidermal thickness, collagen ratio and relative IHC staining index are shown on the right. Data are expressed as mean ± SD (*P < 0.05, **P < 0.01)

To explore whether miR-CM1 also plays a good anti-aging effect at the animal level, the mouse photoaging model was established (Fig. 7a). The treatment group was divided into the miR-Exo group and FELNVs group. The miR-Exo group was treated with the nanoparticles of HA-PEI loaded with miR-CM1 to simulate the exosomes (“artificial exosomes”). HA (hyaluronic acid) in HA-PEI can target the CD44 receptor of skin cells, so HA-PEI has both targeting ability and hydrophilicity and is easy to prepare in large quantities, which can carry and efficiently deliver miR-CM1 to skin cells (Fig. 7b). After 28 days, we collected the dorsal skin of the mice to evaluate the effect of miR-CM1 on UV-induced aging. The dorsal skin of the mice in the model group appeared significantly thickened and loose, while that in the treatment group was only slightly reddened compared with the control group. HE staining results (Fig. 7c) indicated that the model group had disorganized tissue structure, inflammatory cell infiltration, and significantly thickened epidermis, while miR-Exo and FELNVs could significantly alleviate the disorganized skin phenomenon caused by UV irradiation and reduce epidermal thickness. miR-Exo had a better effect than FELNVs in the alleviation of abnormal epidermal hyperplasia. Masson’s staining (Fig. 7d) showed that the loss of dense collagen in the dermal layer was significant in the model group, whereas miR-Exo and FELNVs treatment could significantly increase the collagen content. Immunohistochemistry (Fig. 7e) showed that the expression of Mical2 and MMP1 increased and the expression of COL1A2 decreased in the model group. Compared with the model group, the contents of Mical2 and MMP1 in the treatment group decreased, and the expression of collagen1 increased significantly. The above results indicated that, at the animal level, miR-CM1 could resist the senescence phenomenon induced by UV, and the artificial exosomes encapsulating miR-CM1 could also exert the anti-aging effect like FELNVs extracted from PL.

## Discussion

PL is an important large fungus that normally grows on mulberry trees. As a traditional Chinese medicine, PL has a history of more than 2000 years in eastern countries. Polysaccharides extracted from PL have been shown to have a variety of biological activities, such as anti-inflammatory [9], anti-cancer, hepatoprotective, anti-diabetes, neuroprotective, and anti-angiogenic activities [34]. The water-soluble polysaccharide extracted from PL demonstrates free radical scavenging and antioxidant activities, thereby playing an anti-aging role. At present, the anti-aging mechanism of PL has not been deeply explored. Photoaging accounts for more than 80% of facial aging [35]. UV radiation accelerates skin aging [26]. In this study, we established a UV-induced in vitro and in vivo aging model to study the anti-aging mechanism of PL in depth. We found that FELNVs of PL also had anti-aging effects, which were better than those of water-soluble RNA-free components. Further studies found that miR-CM1 in FELNVs targets 3’UTR of Mical2 and inhibits its expression through cross-species regulation, thereby reducing the levels of ROS, MDA, and SA-β-Gal in skin cells and enhancing SOD activity to play an anti-aging role.

The role of exosomes in the skin has also been reported, for example, Lactobacillus plantarum-derived EVs induce anti-inflammatory M2 macrophage polarization in vitro, regulate skin immunity, and exert anti-inflammatory effects [36]. In this study, we found that FELNVs from PL was related to the anti-aging activity of skin cells. We extracted and characterized PL exosomes (FELNVs) for the first time. The particle sizes of FELNVs are 100-260 nm, similar to those of PELNVs than mammals. Fungal EVs have been reported in the range of 50 to 500 nm in diameter, such as bilayered extracellular vesicles produced by yeast, in the range of 100–300 nm in diameter [20, 37]. Our findings are also consistent with the reported size of fungal EVs. FELNVs of PL have a unique double-layer shell structure. Their unique structure may give it better loading capacity and stability, which needs further study.

Different from mammalian miRNA, the terminal nucleotide of plant-derived miRNA is modified with 2′-O-methyl, a feature that makes it periodate resistant and stable [38]. An increasing number of studies have shown that exogenous plant miRNAs can regulate the expression of mammalian target genes to achieve cross-kingdom regulation [39, 40]. For example, Chenyu Zhang’s team found that miR2911 in honeysuckle is absorbed by mice and inhibits virus replication by directly targeting influenza A virus [41]. miRNAs within PELNVs are highly potent regulators of gene expression and cross-kingdom gene expression regulation. For example, exosomal miRNAs of ginger exosomes can modulate gut flora to improve intestinal health and alleviate colitis in mice [42]. Our work confirmed that miRNAs in the FELNVs of PL had the characteristics of plant-derived miRNAs with 2’-O-methyl modification, which allowed them to be resistant to periodate oxidation. Thus, they could stably target the 3’UTR of Mical2 by cross-kingdom regulation to inhibit its expression and exert anti-aging activity. The shiitake-derived exosome-like nanoparticles discovered by Baolong Liu et al. have protective effects against acute liver injury in mice [43]. Our findings enrich the field of research on fungal EVs, and further studies and demonstrations are needed for more FELNVs from other sources.

Mical2 is a monooxygenase that directly binds and enhances depolymerization of F-actin. In the absence of actin, it also functions as a NADPH oxidase to produce H_2_O_2_. Mical was discovered in Drosophila melanogaster in 2002 [44]. It is involved in the regulation of cytoskeletal dynamics and related basic biological processes [45]. Mical2 is involved in angiogenesis, vesicle transport, and vesicle transport; it is also associated with the occurrence and development of a variety of tumors [46–48]. Our findings suggested that Mical2 was implicated in skin aging. Mical2 could reduce SOD level and accelerate the skin aging process by increasing ROS and SA-β-Gal levels in skin cells.

## Conclusion

We isolated and characterized FELNVs from PL with sizes ranging between 100 and 260 nm. Results showed that FELNVs of PL had anti-aging activity. Our study found five novel miRNAs in FELNVs of PL, among which miR-CM1 had the most obvious anti-aging effect. miR-CM1 plays an anti-aging role by inhibiting the expression of Mical2 in human skin cells through cross-species regulation.

## Methods

### Cell culture

A human immortal keratinocyte line (HaCaT), human skin fibroblasts (HSFs) and human embryonic kidney 293T cells (HEK 293T) were obtained from KeyGEN Biotech (Nanjing, China). Cells were cultured in DMEM (Gibco, USA) supplemented with appropriate fetal bovine serum (FBS) and 100 U/mL penicillin G and grown in 5% moist CO_2_ at 37 °C. HaCaT and HEK 293T cells were supplemented with 10% FBS, while HSF cells were supplemented with 15%.

### Volunteer skin test

Forty volunteers aged 20–35 years participated in this experiment. The 40 volunteers were divided into two groups (n = 20): the control group (treated with basic cream) and the PL group (treated with cream containing 2% PL). Once a day, reagents were smeared on the face and the inside of the arm of the volunteers. The VISIA skin analysis imaging system (Canfield Scientific, USA) was used to record the weekly changes in five indicators (i.e., brown spots, UV spots, wrinkles, speckles, and red zone) for 4 weeks after the use of PL. DermoLab instrument (Cortex Technology, Denmark) was used to detect arm changes in collagen distribution weekly for 4 weeks.

### UV irradiation

After HaCaT cells were pre-protected for 24 h, the original media of the model group and the pre-protection group were discarded and washed with PBS three times. An appropriate amount of PBS was added to each well to cover the cells. The plastic cover was opened, and irradiation was performed at a 10 J/cm^2^ dose using a UV light tube of UVA source (365 nm, PL-S 9 W/10 2P, Philips, Poland). Subsequently, PBS was discarded, fresh medium was replenished, and the cells were cultured until analysis.

For experimental animals, UVA irradiation was performed on a separate area (1.5 cm×1.5 cm) on the back of each mouse in the experimental group at a frequency of three times per week, with a dose of 8 J /cm^2^ for 4 weeks.

### Extraction and identification of FELNVs

FELNVs were isolated following a previous method of ginger-derived nanoparticle (GDNP) isolation with slight modifications [15]. In brief, PL was homogenized in a blender to obtain juice, which was centrifuged at 3,000×g for 30 min to remove dead cells. The supernatant was collected and centrifuged at 10,000×g for 60 min to remove cell debris. The resulting supernatant was further centrifuged at 150,000×g for 90 min, and the pellet was suspended in PBS.

The size and morphology of FELNVs were analyzed by cryo transmission electron microscopy and by using a nanoparticle size analyzer. For morphological identification, an appropriate amount of FELNVs suspended in 20 µL of PBS was loaded onto glow-discharged carbon-coated copper grids. After standing horizontally for 1 min, the filter paper was used to carefully blot the copper grid dry and stained with phosphotungstic acid dye. TEM analysis (Talos L120C G2) was conducted to obtain photos of samples. For particle size analysis, an appropriate amount of FELNVs suspended in 1 mL of PBS was added to the sample cell, and the sample cell was placed into the nanoparticle sizer (Nano-ZS) for testing.

### CCK8 analysis

HaCaT cells were grown in 96-well plates. After pre-protection with PL (low dose 10 µg/mL, medium dose 40 µg/mL, and high dose 100 µg/mL) for 24 h, cells were treated with UVA (10 J/cm^2^) and incubated for an additional 24 h. Cell proliferation was examined using the Cell Counting Kit-8 (ApexBio, MA, USA). A 10 µL CCK8 solution was added to each well in 96-well plates and incubated with cells at 37 °C for 2 h. The absorbance was measured at 450 nm. All the experiments were carried out in triplicate.

### Western blot analysis

The cells were lysed with 1 nM phenyl methyl sulfonyl fluoride (PMSF; Invitrogen, USA) supplemented with RIPA lysis buffer (KeyGEN, Jiangsu, China), and the protein content was determined using a BCA protein assay kit (Thermo Fisher Scientific, USA). For each sample, 10–50 µg of proteins was isolated on 10–15% SDS-PAGE and then transferred to PVDF membrane (Millipore, Bedford). After blocking with 5% skim milk powder, PVDF membrane and primary antibodies against MMP1 (Affinity, 1:1000 dilution), COL1A2 (Stanta, 1:500 dilution), Mical2 (Proteintech, 1:400 dilution), and β-actin (Affinity, 1:1000 dilution) were incubated at 4 °C overnight, and HRP-conjugated secondary antibodies were incubated at 37 °C for 2 h. Imprinting was performed using ECL reagent (Millipore, MA, USA). Protein quality in each lane was identified and standardized using β-actin antibody.

### RNA extraction and quantitative PCR

To extract RNA from HaCaT cells was isolated for homogenization in Trizol (Life Technologies, USA). Total RNA was isolated directly from the cell samples in Trizol. About 3 µg of RNA was reverse transcribed to cDNA by using Quantscript RT kit (Tiangen, China). qRT-PCR analysis was performed using QuantStudio TM 6 (Life Technologies, USA). The sequence of primers is given in Table S1. The expression levels of each gene were normalized to those of β-actin. Each data point contained at least three biological duplicates and is represented as mean ± SD.

For the periodate oxidation assay, the assay was performed according to a previously established method [30]. The total RNA of PL was extracted. About 20 µg of small RNA was co-incubated with 10 mM NaIO_4_ in the dark for 40 min. The samples were precipitated with absolute alcohol twice and rinsed with 80% ethanol once. The samples were then dissolved in DEPC water after air drying, and qRT-PCR assay was performed.

### Immunofluorescence (IF)

Cells were immobilized in 4% paraformaldehyde for 15 min at room temperature and sealed with 5% BSA and 0.03% Triton X-100 for 50 min. After washing with PBS, the cells were incubated with primary antibody against COL1A2 (1:100 dilution) and MMP1 (1:200 dilution) at 4 °C overnight. Alexa Fluor conjugated secondary antibody (Beyotime, Nanjing, China) was incubated at 37 °C for 1.5 h, and the nucleus was stained with DAPI (Beyotime, Nanjing, China). Observations were performed using a laser confocal microscope (Zeiss LSM 800 with Airyscan).

### Skin photoaging mouse model

A total of 24 Kunming mice (female, 6–8 weeks) were housed in a specific pathogen-free animal care facility. Mice were allowed to adapt to the feeding environment for 7 days before the experiment. After the backs were depilated, the mice were randomly divided into four groups (n = 6): control, model (treated with UV), miR-Exo, and FELNVs groups. Mice in the control group were raised normally; the treatment groups were given treatment to the dorsal skin before each UVA irradiation. The miR-Exo group was treated with exosomes mimics (HA-PEI) wrapped with miR-CM1 before each UVA irradiation. The FELNVs group was treated with FELNVs from PL before each UVA irradiation. The model group was treated with placebo before each UVA irradiation. All mice were euthanized after 4 weeks. Dorsal skin tissues were collected and fixed in formalin. Following standard procedures, tissues were dehydrated and embedded in paraffin. Subsequently, 4 μm-thick sections were acquired for hematoxylin eosin (HE) staining, Fontana Masson staining, and immunohistochemical staining.

For HE and Masson staining, according to the manufacturer’s method, Modified HE Stain kit (Solarbio, China) and Modified Masson’s Trichrome Stain Kit (Solarbio, China) were used to evaluate pathological changes of the skin tissues.

### Immunohistochemistry (IHC)

The paraffin-embedded skin tissue was incubated with xylene and gradient ethanol, and the antigens were repaired by boiling the sections in 10 mM citric acid (pH 6.0) for 2 h. The samples were sealed in 1% BSA, 0.3% Triton-x-100, and 10% normal goat serum for 1 h. Endogenous peroxidase was quenched in 3% hydrogen peroxide for 5 min. The samples were incubated overnight at 4 °C with primary antibodies against COL1A2 (1:100 dilution), MMP1 (1:100 dilution), and Mical2 (1:100 dilution). The samples were incubated at 4 °C overnight with the following primary antibodies. After washing with PBS, biotin-conjugated secondary antibody (ZSDB-BIO, China) was used for incubation for 30 min. DAB peroxidase (HRP) substrate kit (ZSDB-BIO, China) was used for color development, and hematoxylin was used for re-staining. The tissue was dehydrated and covered with covered glass (Thermo Scientific, USA).

### Luciferase reporter assay

Luciferase reporter assay was performed to test the inhibitory effect of miR-CM1 on the expression of *MICAL2*, *DUSP18*, *GRAP* and *RRN3* in 293T cells. We cloned the 3’UTRs of these genes individually into the pSiCheck2 vector plasmid. The miR-CM1 mimics or miR-CM1 mutant were cotransfected with constructed pSiCheck2 plasmid into 293T cells. After 48 h of transfection, luciferase activity was detected using a Dual-Lumi™ Luciferase Assay Kit (Beyotime, China).

### Cell function assay

According to the manufacturer’s methods, Lipid Peroxidation MDA Assay Kit (Beyotime, China) was used to determine the levels of MDA, a natural product of lipid oxidation. Senescence β-Galactosidase Staining Kit (Solarbio, China) was used to detect SA-β-gal activity levels. Cu/Zn-SOD and Mn-SOD Assay Kit with WST-8 (Beyotime, China) was used to detect superoxide dismutase (SOD) activity. ROS Assay Kit (Beyotime, China) was used for ROS detection.

### 3D printing

The HSF and HaCaT cells were digested and centrifuged, resuspended with bioink to achieve an HSF and HaCaT cell concentration of 4 × 10^6^ cells/mL, and loaded into a printing needle tube for printing. The printing needle tube mixed with cells was loaded into a cryogenic spray head and further loaded into the printer (Bio-Architect® WS). The number of printed layers in the upper (HaCaT) and lower layers (HSF) was 8 and 4, respectively. The cells were fixed with CaCl_2_ for 10 min, washed with PBS for 2–3 times, added with DMEM, and cultured in a cell incubator at 37 °C with 5% CO_2_. After 7 days of culture, the air–liquid interface was established and replaced with Epi-life medium to promote the formation of stratum corneum. At 21 days, the printed tissues were obtained, embedded with Tissue-Tek O.C.T. Compound (SAKURA, USA), and sliced with a freezer slicer (Leica CM1950) for staining observation.

### Sequencing

miRNA sequencing of exosomes. After FELNVs of PL were obtained by differential UC, total RNA was extracted. Identification of novel miRNAs and their expression differences was performed by miRNA-seq library construction and de novo sequencing.

mRNA sequencing. After transfection of miR-CM1, total RNA was extracted. Gene expression profile changes were obtained by transcriptome sequencing by constructing a poly A-enriched mRNA library.

### Statistical analysis

All final data are presented as mean ± standard deviation (SD). The data were statistically analyzed using the student’s t-test. Significance criteria: *P < 0.05, **P < 0.01.

## Electronic supplementary material

Below is the link to the electronic supplementary material.

Supplementary Material 1

## Data Availability

The datasets used and/or analysed during the current study are available from the corresponding author on reasonable request.
