# Supplementary material for: Exosome-like nanovesicles derived from Phellinus linteus inhibit Mical2 expression through cross-kingdom regulation and inhibit ultraviolet-induced skin aging
Source: J Nanobiotechnology. 2022 Oct 21;20:455. doi: 10.1186/s12951-022-01657-6 (PMC9587628; doi:10.1186/s12951-022-01657-6)
Supplement: Supplementary file 1 — Supplementary Material 1 [file 12951_2022_1657_MOESM1_ESM.docx]

**Supplementary Data**

**Table S1．The primers of RT-PCR.**

| **Gene** | **Primer（5'→3'）** |
| --- | --- |
| MMP1-F | ATGAAGCAGCCCAGATGTGGAG |
| MMP1-R | TGGTCCACATCTGCTCTTGGCA |
| COL1A2-F | CCTGGTGCTAAAGGAGAAAGAGG |
| COL1A2-R | ATCACCACGACTTCCAGCAGGA |
| SHLD1-F | GAGGCTTTCAGTTCTTTGGAATTC |
| SHLD1-R | TTCACAGCAGGGTCAAGCCAGA |
| Mical2-F | TGACAGCCAAGAAGCAGAGCCT |
| Mical2-R | GGTAGTTGGTGGCAAAGTCTGC |
| ZNF383-F | AAAGAGCCCTGGATGGTTGGCA |
| ZNF383-R | GTCCCATTATCTCCCTCTGGCA |
| ITPK1-F | CATCCTTGAAGCCGACCAGAATG |
| ITPK1-R | CTCATAGGACTTGGAGCGGTCA |
| DUSP18-F | ATCCACAGCGTGGAGATGAAGC |
| DUSP18-R | GCGTGGTACTTCATGAGGTAGG |
| GRAP-F | GCCGAAGAGATTCTGATGAAGCG |
| GRAP-R | AAGTGCTGCACCTGGTCTCCAT |
| ACRBP-F | CCTTCAAAAGCCAGCAGTGTCTG |
| ACRBP-R | CTCCATCGAAGCACAACGTCCT |
| PHYHIP-F | CAGGAGAAAGCTGAGCAGATCG |
| PHYHIP-R | GCTGTTGTCCTTCAGGTAAGGC |
| RRN3-F | CGGCAGGGTATTGAAGATGCTG |
| RRN3-R | ACAGGATGCACCATCTGGTCGA |
| HBEGF-F | TGTATCCACGGACCAGCTGCTA |
| HBEGF-R  β-actin-F  β-actin-R | TGCTCCTCCTTGTTTGGTGTGG  CACCATTGGCAATGAGCGGTTC  AGGTCTTTGCGGATGTCCACGT |
| miR-CM1-RT  miR-CM1-F  miR-CM2-RT  miR-CM2-F  miR-CM3-RT  miR-CM3-F  miR-CM4-RT  miR-CM4-F  miR-CM5-RT  miR-CM5-F | GTCGTATCCAGTGCGTGTCGTGGAGTCGGCAATTGCACTGGATACGACTACGTCCA  TGCGGTACCATGGGAGTGGACGTA  GTCGTATCCAGTGCGTGTCGTGGAGTCGGCAATTGCACTGGATACGACTACGGATA  TGCGGTCCTCAAGGTTATCCGTA  GTCGTATCCAGTGCGTGTCGTGGAGTCGGCAATTGCACTGGATACGAC GGCTGTCG  TGCGGCCGGTGCGCTCTCGACAGCC  GTCGTATCCAGTGCGTGTCGTGGAGTCGGCAATTGCACTGGATACGACAACCCGTC  TGCGGACGTGTGGATCCAGACGGGTT  GTCGTATCCAGTGCGTGTCGTGGAGTCGGCAATTGCACTGGATACGACAGGATGGA  TGCGGTATTGGATTCCGTCCATCCT |
| U6-RT  U6-F  Reverse | AACGCTTCACGAATTTGCGT  CTCGCTTCGGCAGCACA  AACGCTTCACGAATTTGCGT |
